# Multidimensional Profiles Associated with Gestational Anemia in Primary Healthcare Networks of Northern Peru

**DOI:** 10.3390/healthcare14142135

**Published:** 2026-07-16

**Authors:** Carola Seijas Ruiz, Maribel Zapata Masias, Magali Tantaleán Pérez

**Affiliations:** 1 Escuela Profesional de Medicina, Facultad de Ciencias de la Salud, Universidad César Vallejo, Trujillo 13001, Peru; caseijasru@ucvvirtual.edu.pe (C.S.R.); zzapatama@ucvvirtual.edu.pe (M.Z.M.); 2 Dirección de Investigación, Universidad César Vallejo, Trujillo 13001, Peru

**Keywords:** gestational anemia, pregnant women, primary health care, sociodemographic factors, multidimensional profiles

## Abstract

**Highlights:**

**What are the main findings?**

- Gestational anemia in northern Peru showed a two-dimensional structure, in which sociodemographic and territorial factors explained a greater proportion of variability than reproductive–biological factors.
- Mild anemia was mainly associated with pregnant women younger than 20 years, lower educational attainment, rural settings, multiparity, and overweight/obesity.
- Moderate/severe anemia was more frequently associated with women aged 20–35 years, secondary or higher educational attainment, urban healthcare settings, primiparity, and normal BMI, contrary to the conventional expectation that higher educational attainment acts as a protective factor against anemia

**What are the implications of the main findings?**

- Recognize gestational anemia as a structural equity issue, not merely as a nutritional deficiency.
- Prenatal care should not be limited to monitoring the current pregnancy. It is essential to systematically inquire about the number of previous pregnancies, the interpregnancy interval, and history of miscarriages, using this information to stratify the risk of anemia from the first visit.
- Studies are needed that directly measure serum levels of hepcidin, ferritin, C-reactive protein, and IL-6 in overweight and obese pregnant women to further explore potential inflammatory mechanisms and evaluate the comparative effectiveness of different supplementation strategies.

**Abstract:**

**Background**: Gestational anemia represents a major public health challenge in Peru, with a national prevalence ranging from 20% to 27.9%; however, the northern regions remain insufficiently studied. This study aimed to explore the multidimensional structure of clinical and sociodemographic characteristics among pregnant women with gestational anemia treated in northern Peru. **Methods**: A cross-sectional study was conducted among 70 pregnant women diagnosed with gestational anemia and treated at the Chicama micro-network in the La Libertad region during 2024. Sociodemographic, territorial, reproductive, and nutritional variables were obtained from medical records. Principal Component Analysis for Categorical Data (CATPCA) was applied to identify multidimensional patterns associated with anemia severity. **Results**: The CATPCA identified a two-dimensional structure explaining 38.47% of the total variance. Dimension 1 (sociodemographic–territorial profile; 22.55%) included health facility (−0.925), educational level (0.887), maternal age (0.878), and district of origin (−0.871). Dimension 2 (reproductive–biological profile; 15.91%) included number of previous pregnancies (0.909), interpregnancy interval (0.909), and body mass index classification (0.589). Multivariate profiles showed that mild anemia (n = 49) was predominantly associated with pregnant women younger than 20 years, lower educational attainment, and rural settings. In contrast, moderate/severe anemia (n = 21) was more frequently associated with women aged 20–35 years, secondary or higher educational attainment, and urban healthcare settings, contrary to the conventional expectation that higher educational attainment acts as a protective factor against anemia. Additionally, mild anemia was associated with multiparity and overweight/obesity, whereas moderate/severe anemia was linked to primiparity and normal BMI. **Conclusions**: Gestational anemia in northern Peru follows a two-dimensional pattern in which sociodemographic and territorial characteristics showed greater explanatory contribution than reproductive–biological factors. Distinct anemia severity profiles were identified, highlighting the need for context-specific prenatal care strategies and targeted public health interventions.

## 1. Introduction

Gestational anemia represents one of the most critical challenges to global public health, characterized by a reduced oxygen-carrying capacity and a shortage of red blood cells, which compromises the mother’s vital functions and fetal development [1]. Its significance lies in the fact that it is a condition associated with high maternal and perinatal morbidity and mortality, in addition to negatively impacting the cognitive and motor development of the newborn, which has long-term consequences for human capital [2,3]. The purpose of this study is to analyze the multidimensional profiles associated with gestational anemia, given its relevance to improving health policies that go beyond a purely biological approach and integrate social, territorial, and economic factors [4,5].

According to the World Health Organization (WHO), anemia during pregnancy affects approximately 36% (32 million) of pregnant women worldwide. This condition, defined by the WHO as a hemoglobin concentration below 11 g/dL in the first and third trimesters, and below 10.5 g/dL in the second trimester, is a key factor in mortality, contributing to more than 115,000 maternal deaths and 591,000 perinatal deaths worldwide each year [6,7,8]. The etiology of this condition is multifactorial; it is not determined solely by biological factors inherent to pregnancy, but rather by a complex interaction with socioeconomic, environmental, prenatal care, lifestyle, and food security factors [9].

Statistically, the burden of the disease is uneven: while prevalence in developed countries is 15%, in developing or underdeveloped countries this figure rises to 48.7% [9]. The most affected regions are Africa (57.1%) and Southeast Asia (48.2%) [9]. In the case of Peru, reports from ENDES and SIEN indicate a national prevalence ranging from 20% to 27.9% [9,10,11]. However, the regional reality is heterogeneous, with departments such as Pasco (37.35%), Puno (33.5%), and Huancavelica (32%) far exceeding the national average [9].

Specifically, a preliminary analysis of the situation in the Chicama micro-network, based on records from the Health Center Information System, reveals an alarming trend. According to preliminary institutional records from the Chicama micro-network Health Information System (HIS), a progressive increase in anemia cases has been observed in recent years, with approximately 70% of diagnosed pregnant women presenting mild anemia and 30% moderate anemia within the local healthcare setting. This local reality, which has not yet been thoroughly documented, underscores the need to explore the multidimensional structure of clinical and sociodemographic characteristics and identify the specific associated factors within this population, which is the rationale for the present study. From a biological standpoint, anemia during pregnancy is primarily due to increased iron requirements for fetal growth, placental development, and the expansion of the maternal red blood cell mass [11,12,13]. This process is accompanied by physiological hemodilution, an increase in plasma volume that reduces the relative concentration of hemoglobin but can lead to pathological anemia if the pregnant woman’s reserves are insufficient [10,14,15].

In the field of obstetrics and reproductive health, multiparity and high multiparity are critical factors, as recurrent pregnancies progressively deplete maternal iron stores [9,13]. Added to this is a short interpregnancy interval (less than two years), which does not allow a woman’s body to restore optimal nutritional levels before a new conception. Furthermore, extreme maternal age, particularly among adolescents and young women aged 18 to 29, represents a high-risk group due to biological immaturity or a lack of knowledge regarding nutritional care [5,16,17].

Sociodemographic factors play a key role in the prevalence of this condition. Poverty and low socioeconomic status limit access to a diverse, micronutrient-rich diet, with food insecurity being a direct predictor of low ferritin levels [9,18,19]. Low educational attainment also acts as a barrier, as it is associated with less knowledge about anemia prevention and the importance of supplementation [4,10,13]. Regarding place of residence, there are discrepancies: while some studies show higher prevalence in rural areas due to a lack of services, others report high rates in urban areas due to economic disparities and poor dietary habits [4,5,20].

Finally, lifestyle and healthcare are key factors. A low pre-pregnancy body mass index (BMI) (underweight) is associated with a higher incidence of anemia, reflecting poor nutritional status prior to pregnancy [3,13]. On the other hand, lack of adherence to ferrous sulfate supplementation and late initiation of prenatal care (or fewer than six visits) hinder timely diagnosis and effective management of the condition, increasing the risk of complications such as low birth weight and preterm birth [3,7,10]. In Peru, antenatal care programs routinely include iron and folic acid supplementation strategies aimed at preventing gestational anemia and reducing maternal and neonatal complications, according to the current national technical guidelines established by the Ministry of Health [21]. However, disparities in access, adherence, and continuity of prenatal care may limit the effectiveness of these preventive interventions, particularly in vulnerable populations [3,10].

Anemia during pregnancy is associated with a number of serious complications for both the mother and the newborn [2,22,23]. In pregnant women, this condition is linked to serious risks such as preterm birth, preeclampsia, placental abruption, and postpartum hemorrhage, the latter being one of the leading causes of maternal mortality worldwide [2,6,9,24]. Additionally, sources indicate a higher frequency of premature rupture of membranes, the need for labor induction, an increase in cesarean sections, greater vulnerability to postpartum infections, and prolonged fatigue [22,25,26]. Studies have also noted its association with an increased risk of postpartum depression [9,15,22]. As for the baby, maternal anemia has been consistently associated with low birth weight, preterm birth, and intrauterine growth restriction [2,3,20].

With regard to the international literature, studies in Ethiopia have identified factors such as young maternal age, heavy menstrual flow, and irregular use of supplements as key associated factors [20]. In Colombia, studies based on the ENSIN highlight the inverse relationship between socioeconomic status and hemoglobin levels, noting that poverty significantly increases the risk [19,27]. In Peru, studies conducted in Southern Lima and Callao associate first-trimester anemia with up to an 11-fold increased risk of low birth weight. Research in Puno and Huancavelica reinforces that multiparity, low educational attainment, and late initiation of prenatal care are critical associated characteristics in the Peruvian population [5,10,13].

The rationale is based on the urgent need to generate local evidence to optimize interventions at the primary care level. Theoretically, the study provides relevant information on how the specific sociocultural and economic associated factors of northern Peru influence the prevalence of anemia, allowing for a better understanding of the issue beyond biological factors. Socially, the findings align with Sustainable Development Goal (SDG) 3, specifically with the targets for reducing maternal and neonatal mortality. From a practical perspective, the results will enable managers of the Chicama micro-network to redesign strategies for nutritional counseling and prenatal follow-up, ensuring more effective iron supplementation tailored to the realities of the vulnerable population [17,19,28].

In this context, the main purpose of this article is to identify the multidimensional profiles associated with gestational anemia in pregnant women receiving care at a primary healthcare micro-network in northern Peru. This objective is strategically aligned with SDG 3 (Good Health and Well-being), particularly with targets 3.1 and 3.2, which aim to reduce maternal and neonatal mortality. By identifying the multidimensional profiles associated with gestational anemia, this study contributes to strengthening preventive prenatal care at the primary care level, thereby reducing critical risks such as postpartum hemorrhage and preterm birth key factors for the survival of the mother–child pair [29].

## 2. Materials and Methods

### 2.1. Type, Approach, and Design of the Research

The study is classified as basic research according to the Frascati Manual [30], aimed at generating knowledge about the underlying causes of observable phenomena that have not yet been explained, with an emphasis on understanding the associated factors and multidimensional profiles associated with gestational anemia in pregnant women. A quantitative approach was adopted, which allowed for the collection and analysis of numerical data including age, weight, hemoglobin levels, and the number of prenatal visits using statistical procedures to obtain objective and potentially generalizable conclusions. The study is observational rather than experimental, as the variables were analyzed as they occurred in reality without active intervention, using retrospective information from medical records and HIS records corresponding to the year 2024. Its design is cross-sectional, with an explanatory–analytical scope [28]. This scope is relevant because the research is not limited to describing the frequency of anemia or identifying bivariate associations, but rather seeks to establish the underlying multidimensional structure of the associated characteristics using multivariate analysis techniques such as Categorical Autocorrelation Principal Component Analysis (CATPCA). Thus, the explanatory design aligns with the objective of understanding how the multidimensional profiles associated with gestational anemia are organized and related in the study population, beyond mere description or correlation [28].

### 2.2. Variables

In this study, the dependent variable was gestational anemia, defined as a hemoglobin concentration of less than 11 g/dL in the first and third trimesters, and below 10.5 g/dL in the second trimester, according to World Health Organization criteria. This leads to a reduction in the blood’s oxygen-carrying capacity, affecting the physiological needs of both the mother and the developing fetus [11,17,18,29]. Data were obtained from the institutional health information system. Hemoglobin levels were specifically extracted from the records of the first prenatal check-up, predominantly occurring during the first trimester of pregnancy. This allowed for a baseline assessment of the participants’ hematological status. Anemia was defined according to the World Health Organization and Peruvian national protocols (hemoglobin < 11.0 g/dL, adjusted for altitude), regardless of the specific underlying etiology, as ferritin levels or other inflammatory biomarkers were not available in the routine primary care records. The independent variable corresponded to the associated characteristics linked to the multidimensional profiles associated with gestational anemia, including: sociodemographic factors (maternal age, marital status, educational level, and primary occupation); reproductive and biological factors (gestational trimester, number of previous pregnancies, number of previous abortions, prenatal check-ups, and interpregnancy interval); nutritional factors (pre-pregnancy BMI and type of supplementation); and complications during pregnancy [9,13,24,31,32,33,34,35].

### 2.3. Population and Sampling

#### 2.3.1. Population

The study population consisted of 70 pregnant women with a confirmed diagnosis of gestational anemia including mild, moderate, and severe forms who constituted the unit of analysis [36]. To ensure data integrity and the internal validity of the results, strict eligibility criteria were applied. Inclusion criteria included a confirmed diagnosis of anemia, complete documentation of information in the medical record, and regular attendance at prenatal check-ups. On the other hand, medical records that were illegible or missing key variables were excluded, as were pregnant women with preexisting medical conditions (hematological, chronic, or infectious) that could act as confounding factors in the relationship between the associated characteristics and anemia severity profiles within the studied population [37]. Overweight and obesity alone were not considered exclusion criteria, as these conditions were analyzed as nutritional characteristics within the multidimensional profiles associated with gestational anemia.

#### 2.3.2. Sampling

A census-style sampling method was chosen, as it included all members of the population who met the criteria of interest within the defined time frame and context. This strategy is particularly suitable when working with finite and accessible populations, allowing for a comprehensive characterization of the phenomenon without the need to calculate sample sizes or account for sampling errors resulting from randomization [37].

By analyzing all available cases (n = 70), we ensure maximum representativeness within the micro-network and reduce the risk of selection bias, thereby strengthening the credibility of the inferences regarding the multidimensional profiles associated with gestational anemia in this specific primary care setting [37].

### 2.4. Data Collection Techniques

To collect the data, techniques and instruments consistent with the quantitative approach and the nature of the variables associated with gestational anemia were selected. The primary technique was secondary data analysis (documentary verification), based on a review of medical records from the Chicama micro-network. For this purpose, an ad hoc data collection form was designed, an instrument that allowed for the systematic recording and quantification of the operationalized variables, associated with the multidimensional profiles associated with gestational anemia. To ensure that the instrument accurately represented the domain of the variables under study, it underwent face validity testing; in this process, specialists in obstetrics and gynecology, public health, and methodology evaluated the clarity, comprehensiveness, and relevance of each item, providing solid evidence of its content validity [28].

In addition, a pilot study was conducted with a small segment of the population to test methodological and logistical aspects of the research. The pilot phase included the review of 20 medical records selected from the same healthcare micro-network. This preliminary study made it possible to verify the applicability of the questionnaire, estimate the time required for data collection, and identify potential ambiguities in the interpretation of the records. Based on the pilot evaluation, minor adjustments were made to standardize variable coding and improve consistency in data extraction procedures, thereby avoiding flaws that could compromise the quality of the main study [38]. The objectivity and standardization of the process were ensured through training on data extraction criteria, and adjustments derived from the pilot study were incorporated into the final version of the instrument to ensure that the collected data were accurate, consistent, and valid [28].

### 2.5. Data Analysis

Once the data had been collected, it was processed and analyzed using statistical techniques appropriate for the quantitative approach and the descriptive design of the study. First, the information from the forms was entered into the Statistical Package for the Social Sciences (SPSS) version 30 to create a database, clean it, and handle missing or outlier values, thereby ensuring the quality of the data. Next, a descriptive analysis was conducted to summarize and characterize the data from the study population. For the quantitative variables (maternal age, number of pregnancies, and pre-pregnancy body mass index), descriptive measures were used, while for the qualitative variables (marital status, educational level, health facility, among others), absolute and relative frequency distributions were presented [28,39].

To explore the underlying structure of categorical variables and their relationship with gestational anemia, a Principal Component Analysis for Categorical Data (CATPCA) was used. This method allowed the identification of latent dimensions that explain the joint variability of sociodemographic, territorial, and reproductive–biological associated factors, as well associated factors. Furthermore, this technique reduces the dimensionality of a set of nominal or ordinal variables through the optimal quantification of categories. Two dimensions were retained based on the criterion of eigenvalues greater than 1 and the interpretability of the solutions.

CATPCA was specifically chosen to explore latent multidimensional structures and profiles among pregnant women with anemia, rather than to identify independent clinical predictors. This approach allowed the integration of sociodemographic, territorial, reproductive, and biological characteristics into clinically interpretable severity profiles.

The model explained 38.47% of the total variance (22.55% for the first dimension and 15.91% for the second). According to Hair et al. (2019) [40], in social and health research where data are categorical, this range is acceptable. Although the sample size was relatively small (n = 70), the study included the entire accessible population of pregnant women diagnosed with anemia within the study period, consistent with a census-based approach. CATPCA was selected because it is suitable for exploratory dimensional reduction in categorical datasets and for identifying latent multidimensional structures in health research contexts. Therefore, the findings should be interpreted as exploratory multidimensional profiles rather than confirmatory population-level models to ensure a simplified yet valid representation of the phenomenon, given that the variance captured tends to be lower than in traditional linear models. With adequate reliability (total Cronbach’s alpha = 0.906) the factor loadings and category scores allowed for the identification of two clear multidimensional structures in the data: the first dimension grouped sociodemographic and territorial variables (maternal age, educational level, health facility, district of origin), and was primarily associated with anemia severity profiles; the second dimension included variables related to reproductive and biological profiles (number of previous abortions, number of previous pregnancies, interpregnancy interval, and BMI classification). The results are presented in tables and category-based graphs that facilitate the interpretation of the relationships between the variables [28,39].

### 2.6. Ethical Considerations

This study was conducted in strict accordance with the ethical principles set forth in the Declaration of Helsinki and Peru’s General Health Law (Law No. 26842) [39,41]. It was approved by the Research Ethics Committee of the Faculty of Medicine at César Vallejo University (Approval No. 00501-2025/CEI-EPM on 25 June 2025). Data were obtained from medical records, with prior authorization from the facilities in the Chicama micro-network, and were handled with due confidentiality through the coding of records and databases, protecting the participants’ identities by avoiding the use of names or direct identifiers in both the analysis and the dissemination of the results. All information collected was used exclusively for scientific purposes, ensuring that plagiarism or falsification was prevented through proper citation of sources. Throughout the process, a rigorous methodology based on validated scientific methods was applied, ensuring accuracy in the conduct of the study. Data analysis was conducted objectively and impartially, preventing biases, ideologies, or external interests from influencing the interpretation of the findings. The interpretation of the results was limited to the identification of multidimensional profiles and associated characteristics within the studied anemic population, avoiding causal inferences beyond the scope of the study design. Finally, transparency in the communication of results was ensured through a detailed description of statistical procedures, explicit disclosure of potential conflicts of interest, proper citation of sources, and acceptance of responsibility for the integrity of the academic work [39,41].

## 3. Results

To achieve the overall objective, the following specific objectives were established:

### 3.1. Objectively Identify the Multidimensional Profiles Associated with Gestational Anemia Among Pregnant Women Diagnosed with Anemia

Table 1: These results revealed the existence of two distinct profiles of pregnant women: one of a socio-educational and territorial nature linked to anemia, and another of a reproductive and obstetric nature associated with pregnancy history and nutritional status; the variables with the greatest contribution to Dimension 1 (sociodemographic–territorial) were the health facility (−0.925), educational level (0.887), maternal age (0.878), and district of origin (−0.871). The classification of anemia also showed a moderate loading in this dimension (−0.511), indicating that anemia severity showed a stronger association with social and geographic characteristics within the analyzed population. Meanwhile, Dimension 2 (reproductive–biological) was primarily characterized by the number of previous pregnancies (0.909), interpregnancy interval (0.909), number of abortions (0.616), BMI classification (0.589), and primary occupation (−0.522), reflecting differences in obstetric history, nutritional status, and socioeconomic activity profiles within the studied population rather than direct differentiation of anemia severity.

### 3.2. Identify the Sociodemographic, Geographic, and Reproductive Factors Associated with Mild and Moderate/Severe Anemia

Table 2 shows a clear differentiation of anemia severity profiles within Dimension 1 (sociodemographic–territorial). The mild anemia category presented a positive coordinate (0.335), whereas moderate/severe anemia showed a negative coordinate (−0.781). This distribution suggests that sociodemographic and territorial characteristics were the principal elements differentiating anemia severity profiles among the studied pregnant women. A consistent pattern was identified in relation to the analyzed variables. Pregnant women with mild anemia tended to present profiles characterized by being under 20 years of age (coordinate −1.014), having no education or only primary education (coordinates between −0.620 and −0.955), receiving care at the Alto Perú or CSMI Chicama healthcare facilities (coordinates between 0.559 and 1.014), and residing in the Chicama district (coordinate 1.017). In contrast, moderate/severe anemia profiles were more frequently associated with women aged 20–35 years or older (coordinates 0.935 and 0.059, respectively), secondary or higher educational attainment (coordinates between 0.588 and 0.997), care at the Santa Rosa or Santiago de Cao facilities (coordinates between −0.941 and −0.938), and urban or peri-urban areas such as Santiago de Cao (coordinates between −1.107 and 0.045).

Table 3 presents the reproductive–biological characteristics associated with anemia severity profiles within Dimension 2 (reproductive–biological). Unlike Dimension 1, the variables grouped in this dimension did not directly differentiate anemia severity but instead reflected distinct obstetric and nutritional profiles within the studied population. Pregnant women with mild anemia tended to present profiles characterized by multiparity (coordinate 0.962), an interpregnancy interval of 24 months or more (coordinate 0.956), at least one previous abortion (coordinate 1.231), and overweight or obesity (coordinates between −0.364 and 0.506). In contrast, moderate/severe anemia profiles were more frequently associated with primigravida women (coordinate −0.858), absence of previous abortions (coordinate −0.308), normal BMI (coordinate −1.202), and cases in which the interpregnancy interval was not applicable because it corresponded to a first pregnancy. Overall, these findings suggest that reproductive and biological characteristics contributed to the multidimensional characterization of anemia severity profiles, complementing the sociodemographic–territorial patterns identified in Dimension 1.

Taken together, the findings from Table 2 and Table 3 suggest the presence of two complementary multidimensional structures associated with anemia severity profiles among pregnant women in the studied micro-network. The sociodemographic–territorial dimension showed a clearer differentiation between mild and moderate/severe anemia profiles, whereas the reproductive–biological dimension contributed additional characterization related to obstetric history and nutritional status. These results reinforce the multidimensional nature of gestational anemia within the studied population and highlight the importance of considering both contextual and reproductive characteristics in prenatal care assessment.

Table 3 shows that the CATPCA identified two principal dimensions that account for 38.47% of the total variability in the data. The first dimension (22.55%) reflected sociodemographic and territorial characteristics, including maternal age, educational level, health facility, and district of origin, as well as the level of anemia. The second dimension (15.91%) reflected reproductive and biological characteristics, such as number of previous pregnancies, interpregnancy interval, number of abortions, and BMI classification.

## 4. Discussion

An analysis of Table 1 reveals two critical dimensions underlying the multidimensional profiles associated with gestational anemia within the studied population: a sociodemographic–territorial dimension (Dimension 1) and a reproductive–biological dimension (Dimension 2).

In Dimension 1 (sociodemographic–territorial), healthcare facility (−0.925), educational attainment (0.887), maternal age (0.878), and district of origin showed the highest factor loadings, suggesting that anemia severity profiles may be more strongly associated with contextual and territorial characteristics than with isolated biological factors. The strong influence of healthcare facilities aligns with findings reported by Díaz-Tinoco and Munares-García, who described a higher prevalence of anemia in MINSA healthcare centers compared with private facilities [42]. Likewise, educational attainment has consistently been associated with differences in nutritional knowledge, adherence to supplementation, and access to diverse diets in studies conducted in Pakistan, Ghana, and Peru [43,44,45,46]. Maternal age also emerged as a relevant characteristic, consistent with evidence identifying adolescents and women of advanced maternal age as vulnerable groups due to biological immaturity or progressive depletion of nutritional reserves [5,13,44,46,47]. Furthermore, territorial disparities between healthcare facilities and districts are consistent with previous reports describing differences in healthcare access and nutritional conditions between urban and rural populations [45,46,47]. From a clinical perspective, these multidimensional profiles may help primary healthcare services identify groups of pregnant women requiring differentiated nutritional monitoring and prenatal follow-up strategies.

In Dimension 2 (reproductive–biological), the number of previous pregnancies (0.909), interpregnancy interval (0.909), number of previous abortions (0.616), BMI classification (0.589), and primary occupation (−0.522) contributed to the reproductive–biological structure identified by the CATPCA model. These findings are consistent with literature describing multiparity and short interpregnancy intervals as characteristics associated with progressive depletion of maternal iron stores [5,16,31,48]. Abdilahi et al. similarly reported a high proportion of anemia among multiparous pregnant women [31]. Primary occupation also contributed moderately to this dimension, suggesting that occupational and socioeconomic conditions may influence the reproductive–biological profiles observed among pregnant women with anemia. In addition, BMI variability may reflect nutritional heterogeneity within the studied population, since both undernutrition and obesity have been associated with alterations in iron metabolism through different physiological pathways [9,13].

Finally, the moderate loading of anemia classification within the sociodemographic dimension (−0.511) suggests that anemia severity profiles may be associated with environmental, educational, and territorial characteristics within the studied population. These findings support the multidimensional nature of gestational anemia and highlight the potential importance of integrated prenatal care strategies that combine nutritional monitoring with improved healthcare access and educational support [26,42]. Within the Peruvian primary healthcare system, maternal vaccination against influenza and Tdap is routinely incorporated into prenatal care as part of maternal preventive strategies, although maternal RSV vaccination has not yet been widely implemented in public healthcare services. Likewise, oral ferrous sulfate combined with folic acid remains the standard strategy for the prevention and treatment of gestational iron-deficiency anemia. Although information regarding iron supplementation was available in the reviewed medical records, vaccination status, adherence to treatment, and exact treatment duration were not consistently documented, limiting their inclusion in the present analysis [49].

The findings presented in Table 2 suggest that anemia severity profiles among the studied pregnant women were primarily differentiated by sociodemographic and territorial characteristics. Mild anemia was predominantly associated with pregnant women under 20 years of age and with lower educational attainment. This vulnerability among adolescents is consistent with studies conducted in Cuba [16] and with evidence from the ENDES in Peru, which identifies this group as highly vulnerable due to the competition between maternal growth requirements and fetal development [5,9,50]. Likewise, low educational attainment may limit knowledge regarding nutritional supplementation and dietary diversity, contributing to persistent anemia profiles [45,46].

In contrast, moderate/severe anemia profiles were more frequently associated with women aged 20–35 years or older, secondary or higher educational attainment, and urban or peri-urban healthcare settings such as Santiago de Cao. Although educational level is traditionally considered a protective characteristic against anemia, similar paradoxical findings have been described in urban populations where dietary transitions and inconsistent adherence to supplementation may persist despite greater educational access [33,43,51]. Karami et al. (2022) [11] similarly noted that greater nutritional knowledge does not necessarily translate into healthier dietary practices in urban settings.

However, an alternative explanation may involve detection bias, since women with higher educational attainment and those attending urban healthcare facilities may receive more comprehensive prenatal follow-up and laboratory screening, increasing the likelihood of identifying and classifying moderate/severe anemia. Therefore, the observed association should be interpreted cautiously within the context of healthcare access and diagnostic practices.

Likewise, the geographical disparity observed between healthcare facilities and districts suggests that territorial context may contribute to the multidimensional characterization of anemia severity profiles. Differences in referral dynamics, healthcare complexity, and access to prenatal monitoring between urban and rural healthcare centers may partially explain the greater identification of moderate/severe anemia cases in urban facilities, whereas milder cases in rural settings could remain underdiagnosed [4,11,52]. Overall, these findings suggest that anemia severity profiles within the studied micro-network may be influenced by territorial context, healthcare access, and sociodemographic characteristics, highlighting the potential importance of context-specific prenatal monitoring and nutritional counseling strategies.

The findings presented in Table 3 suggest that reproductive and biological characteristics contributed to the multidimensional characterization of anemia severity profiles among the studied pregnant women. Mild anemia profiles were more frequently associated with multiparity, previous abortions, interpregnancy intervals of 24 months or more, and overweight or obesity. The association with multiparity is consistent with previous literature describing the progressive depletion of maternal iron stores due to successive pregnancies, particularly when nutritional recovery between pregnancies is insufficient [9,31,53]. However, unlike studies conducted in some African populations, where multiparity has been more strongly associated with severe anemia, the present findings suggest that within this population multiparity may be linked to milder anemia profiles, possibly reflecting differences in healthcare access, nutritional support, or reproductive patterns.

The association between mild anemia and overweight/obesity may be consistent with the anemia-inflammation hypothesis described in previous studies, in which obesity, as a chronic low grade inflammatory state, may alter iron metabolism through cytokine mediated pathways involving hepcidin regulation [9,13,54,55,56,57]. However, ferritin and inflammatory biomarkers were unavailable in the present study. Therefore, the observed pattern may reflect possible alterations in iron metabolism associated with adipose tissue rather than a confirmed inflammatory subtype of anemia. In contrast, moderate/severe anemia profiles were more frequently associated with primigravida women, normal BMI, and the absence of previous abortions. This pattern may suggest a preexisting absolute iron deficiency among women entering pregnancy with limited iron reserves. Previous studies indicate that many women begin pregnancy with depleted iron stores due to menstrual blood loss, inadequate dietary intake, or insufficient preconception nutritional preparation [5,9,13,54,58]. In addition, primigravida women may have less prior experience with prenatal supplementation and nutritional care, potentially increasing their vulnerability to more severe anemia during pregnancy.

Overall, these findings suggest that reproductive and nutritional characteristics may contribute to different anemia severity profiles among pregnant women in the studied micro-network. These multidimensional reproductive–biological patterns complement the sociodemographic–territorial profiles identified in Dimension 1 and highlight the potential importance of individualized prenatal nutritional assessment according to reproductive history and nutritional status. Table 4 demonstrates that the CATPCA model showed acceptable internal consistency and exploratory capacity for identifying multidimensional profiles associated with gestational anemia within the studied population. Dimension 1 (sociodemographic–territorial) explained the largest proportion of variance (22.55%), suggesting that contextual and territorial characteristics contributed substantially to the differentiation of anemia severity profiles. Dimension 2 (reproductive–biological) accounted for an additional 15.91% of the explained variance, indicating that reproductive history and nutritional characteristics provided complementary information within the multidimensional structure identified by the model.

The overall Cronbach’s alpha (0.906) indicates excellent internal consistency for the CATPCA dimensions as a whole, supporting the reliability of the exploratory multidimensional structure observed in this study. Although the explained variance was moderate, this proportion is considered acceptable in categorical exploratory analyses involving complex health-related variables. Therefore, the findings should be interpreted as exploratory multidimensional profiles rather than confirmatory population-level models.

Overall, these findings suggest that gestational anemia in studied Peruvian population may be influenced by the interaction of sociodemographic, territorial, reproductive, and nutritional characteristics beyond exclusively biological factors, highlighting the potential importance of context-specific prenatal care strategies [42].

### 4.1. Practical Implications

Gestational anemia in Peru constitutes a complex phenomenon that transcends the clinical sphere and is closely linked to structural, territorial, educational, and reproductive factors associated with different clinical profiles of gestational anemia. The marked variability in the quality of care across different healthcare facilities highlights that this is not merely an individual problem of the pregnant woman, but a reflection of gaps within the healthcare system.

In this sense, it is essential to standardize screening, supplementation, and nutritional follow-up processes, while also ensuring equitable access to services particularly in rural areas where the risk of underdiagnosis is significantly higher.

Reproductive history emerges as an underutilized clinical marker in clinical practice. Multiparity and short interpregnancy intervals were associated with more severe anemia profiles in the studied population, supporting the importance of incorporating obstetric history into prenatal risk stratification. Consequently, pregnant women presenting with these characteristics may require closer nutritional and hematological monitoring during pregnancy.

In the educational field, the presence of moderate and severe anemia in women with secondary or higher education calls into question the idea that formal schooling acts as a protective factor. This finding may indicate the importance of strengthening nutritional health literacy, focusing on effective preventive practices that go beyond mere years of formal instruction.

From a nutritional dimension, the association between mild anemia and overweight or obesity reveals an inflammatory subtype mediated by hepcidin, where adipose tissue limits iron absorption. This necessitates the design of differentiated strategies that include the management of the inflammatory state, given that conventional supplementation protocols may prove insufficient for this specific group.

Finally, the concentration of moderate and severe anemia in primigravida women with normal weight highlights the importance of pre-pregnancy iron stores. This finding supports the relevance of early nutritional assessment and timely supplementation before fetal demand intensifies iron deficiency during the second and third trimesters.

Taken together, this evidence supports a comprehensive and segmented approach that acknowledges the heterogeneity of the multidimensional profiles associated with gestational anemia. Public policies aimed at reducing its burden should move beyond mass iron supplementation and advance toward structural interventions that guarantee equity in the quality of prenatal care, effective nutritional education, and differentiated strategies according to the identified clinical and sociodemographic profiles. Future studies with larger samples and non-anemic comparison groups could incorporate regression-based models to evaluate independent associations and strengthen clinical prediction.

### 4.2. Limitations and Future Research

This study has several limitations that must be taken into account when interpreting its results. In the first place, its cross-sectional design does not allow for the establishment of causal relationships between the identified factors and the onset of anemia during pregnancy. Although the factor analysis made it possible to identify critical dimensions that link the associated factors, it is not possible to determine the temporal direction of these associations, nor to establish with certainty whether the sociodemographic–territorial and reproductive–biological determinants precede the anemic state. Additionally, because the study included only pregnant women diagnosed with anemia and did not incorporate a non-anemic comparison group, the findings should not be interpreted as causal determinants or risk factors for the development of gestational anemia. Instead, the results reflect multidimensional profiles and associations within an already anemic population.

Secondly, the study is limited to a primary care micro-network in northern Peru, which restricts the generalizability of the findings to other regional realities. As evidenced by the discrepancies with studies conducted in Puno where biological factors associated with altitude outweigh educational ones and in Lima where urban areas show anemia rates linked to lifestyles that promote anemia, the multidimensional profiles associated with gestational anemia can vary significantly depending on the geographic and sociocultural context, as well as the organization of health services. Therefore, the results obtained represent the specific reality of the northern Peruvian region under study but are not necessarily extrapolable to other regions of the country. Furthermore, differences in referral dynamics, diagnostic capacity, and prenatal monitoring among health centers may have influenced the observed distribution of anemia severity profiles between urban and rural centers.

Thirdly, the research was based on medical records and institutional databases, which introduces limitations inherent to the quality and completeness of secondary information. Relevant variables such as actual adherence to iron supplementation, the frequency and timing of prenatal check-ups, or the presence of comorbidities like chronic infections or intestinal parasites could not be included in the analysis due to a lack of systematic recording in the consulted sources. Likewise, information regarding maternal vaccination status against influenza, Tdap, or RSV, as well as the exact duration of iron treatment, was not consistently available in all reviewed clinical records. Furthermore, information regarding serum levels of hepcidin, ferritin, or inflammatory markers was unavailable, which would have allowed for a deeper exploration of the pathophysiological mechanisms of anemia associated with overweight and obesity. A limitation of this study is the lack of biomarkers such as serum ferritin and inflammatory indicators, which prevented differentiation between iron deficiency anemia and other potential types of anemia. Furthermore, since hemoglobin was measured at a single point during early prenatal assessment, the longitudinal progression of anemia throughout pregnancy could not be evaluated.

Fourthly, while the applied CATPCA model identified a bidimensional structure of the multidimensional profiles associated with gestational anemia, the explanatory power of the model showed differences between dimensions, with an explained variance of 22.55% in Dimension 1 and 15.91% in Dimension 2. Although the sample size was relatively small (n = 70), the study included the entire accessible population of pregnant women diagnosed with anemia during the study period, consistent with a census-based approach. CATPCA was selected because it is suitable for exploratory dimensional reduction in categorical datasets and for identifying latent multidimensional structures in health research contexts. Therefore, the findings should be interpreted cautiously as exploratory multidimensional profiles rather than confirmatory population-level models. Additionally, the relatively small sample size may limit the statistical stability and external reproducibility of the CATPCA model. Other factors not included in the analysis such as the quality of nutritional counseling during prenatal care, household food security, family support, or pre-pregnancy anemia may also contribute to the observed variability and should be explored in future multicenter studies with larger populations.

## 5. Conclusions

Gestational anemia in northern Peru appears to follow a bidimensional pattern in which sociodemographic and territorial characteristics showed a greater explanatory contribution (22.55%) than reproductive–biological factors (15.91%). The findings suggest the presence of multidimensional anemia severity profiles associated with health facilities characteristics, educational attainment, territorial context and reproductive history within the studied population. Accordingly, the results indicate that prenatal care strategies may benefit from approaches combining nutritional counseling, early screening, and context-specific follow-up beyond iron supplementation alone.

The anemia severity profiles observed in this population challenge conventional expectations by showing that moderate/severe anemia was more frequently identified among women with secondary or higher educational attainment and from urban healthcare settings. These findings may partially reflect differences in healthcare access, referral dynamics, prenatal monitoring practices, or diagnostic detection across healthcare facilities. Furthermore, two contrasting reproductive–biological profiles were identified: one associated with overweight/obesity in multiparous women and another associated with primiparous women with normal BMI. However, because ferritin and inflammatory biomarkers were unavailable, these profiles should be interpreted cautiously and not as definitive pathophysiological classifications.

Overall, the findings suggest that anemia severity profiles within a population may be influenced by the interaction of territorial, healthcare-related, reproductive, and nutritional characteristics. Therefore, the study highlights the potential importance of equity-oriented and context-specific prenatal care strategies aimed at strengthening nutritional counseling, prenatal follow-up, and individualized maternal support according to reproductive and nutritional profiles.

## Figures and Tables

**Table 1:** Factor loadings and contributions to the dimensions of the CATPCA model.

Variable	Dimension 1 (Sociodemographic–Territorial)	Dimension 2 (Reproductive–Biological)
Healthcare facility	**−0.925**	0.085
Level of education	**0.887**	−0.046
Mother’s age	**0.878**	−0.123
District of origin	**−0.871**	0.104
Number of previous pregnancies	0.066	**0.909**
Intergenetic period	0.066	**0.909**
Number of previous abortions	0.126	**0.616**
IMC Classification	−0.037	**0.589**
Primary occupation	−0.096	**−0.522**
Classification of Anemia	−0.511	**−0.043**

Note: Bold values indicate factor loadings ≥ |0.50|, considered to have a high contribution to the respective dimension.

**Table 2:** Sociodemographic–territorial profiles associated with anemia severity.

Variable	Category	Coordinate Dimension 1 Sociodemographic–Territorial	Association with Anemia	Frequency
Anemia Classification	Mild anemia (Hb 10.0–10.9 g/dL)	0.335	–	49
	Moderate/severe anemia (Hb < 10.0 g/dL)	−0.781	–	21
Mother’s age	under 20	−1.014	Mild	30
	20–35 years old	0.935	Moderate/severe anemia	32
	>35 years old	0.059	Moderate/severe anemia	8
Level of education	None/Elementary	−0.620 to −0.955	Mild anemia	36
	High School/Advanced Technical School	0.588 to 0.997	Moderate/severe anemia	34
Healthcare facility	Alto Perú/CSMI Chicama	0.559 to 1.014	Mild anemia	36
	Santa Rosa/Santiago the Cao	−0.941 to −0.938	Moderate/severe anemia	34
District of origin	Chicama	1.017	Mild anemia	24
	Santiago de Cao/Other	−1.107 to 0.045	Moderate/severe anemia	46

Note: The coordinates correspond to the category quantifications within Dimension 1 (sociodemographic–territorial) of the CATPCA model. Negative and positive values indicate the relative position of each category within the perceptual space. Associations with mild or moderate/severe anemia were derived from spatial proximity and category distribution patterns.

**Table 3:** Reproductive–biological profiles associated with anemia severity.

Variable	Category	Coordinate Dimension 2 (Reproductive–Biological)	Associated Severity Profile	Frequency
Number of previous pregnancies	0 (primigravida)	−0.858	Moderate/severe anemia	37
	≥1 (previous pregnancy)	0.962	Mild anemia	33
Interpregnancy interval	Not applicable (first pregnancy)	−0.858	Moderate/severe anemia	37
	≥24 months	0.956	Mild anemia	29
Number of previous abortions	0	−0.308	Moderate/severe anemia	56
	≥1	1.231	Mild anemia	14
BMI Classification	Normal	−1.202	Moderate/severe anemia	8
	Overweight/Obesity	−0.364 to 0.506	Mild anemia	62

Note: the coordinates correspond to the category quantifications within dimension 2 (reproductive–biological) of the CATPCA model. Negative and positive values indicate the relative position of each category within the perceptual space. Associations with mild or moderate/severe anemia were derived from spatial proximity and category distribution patterns. The coordinate range observed for overweight/obesity reflects variability between grouped BMI categories included in the analysis.

**Table 4:** CATPCA model quality.

Dimension	Cronbach’s Alpha	Self-Worth	Percentage of Variance Explained	Interpretation
Dimension 1 (sociodemographic–territorial)	0.798	4.059	22.55	Good internal consistency; explains the largest proportion of variance
Dimension 2 (reproductive–biological)	0.689	2.864	15.91	Acceptable consistency; explains significant additional variance
Total/Average	0.906	6.924	38.47	Excellent overall model consistency

Note: In CATPCA, values of explained variance between 30% and 50% are considered acceptable for complex categorical data. The overall Cronbach’s alpha of 0.906 indicates excellent reliability for the dimensions as a whole.

## Data Availability

The data supporting the findings of this study are not publicly available due to ethical and privacy restrictions involving sensitive participant information. Relevant anonymized data supporting the conclusions of this article are included within the article. Further inquiries may be directed to the corresponding author.

## Abbreviations

The following abbreviations are used in this manuscript:

SDG 3	Sustainable Development Goal 3
WHO	World Health Organization
MINSA	Ministerio de Salud (Acronyms in Spanish)
